# A Comprehensive Assessment of the Secretome Responsible for Host Adaptation of the Legume Root Pathogen *Aphanomyces euteiches*

**DOI:** 10.3390/jof8010088

**Published:** 2022-01-17

**Authors:** Andrei Kiselev, Hélène San Clemente, Laurent Camborde, Bernard Dumas, Elodie Gaulin

**Affiliations:** Laboratoire de Recherche en Sciences Végétales (LRSV), Université de Toulouse, CNRS, UPS, Toulouse INP, 31320 Toulouse, France; kiselev@lrsv.ups-tlse.fr (A.K.); sancle@lrsv.ups-tlse.fr (H.S.C.); camborde@lrsv.ups-tlse.fr (L.C.); dumas@lrsv.ups-tlse.fr (B.D.)

**Keywords:** oomycete, *Aphanomyces*, secretome, effector, legume, adaptation, SSP, pathogenicity

## Abstract

The soil-borne oomycete pathogen *Aphanomyces euteiches* causes devastating root rot diseases in legumes such as pea and alfalfa. The different pathotypes of *A. euteiches* have been shown to exhibit differential quantitative virulence, but the molecular basis of host adaptation has not yet been clarified. Here, we re-sequenced a pea field reference strain of *A. euteiches* ATCC201684 with PacBio long-reads and took advantage of the technology to generate the mitochondrial genome. We identified that the secretome of *A. euteiches* is characterized by a large portfolio of secreted proteases and carbohydrate-active enzymes (CAZymes). We performed Illumina sequencing of four strains of *A. euteiches* with contrasted specificity to pea or alfalfa and found in different geographical areas. Comparative analysis showed that the core secretome is largely represented by CAZymes and proteases. The specific secretome is mainly composed of a large set of small, secreted proteins (SSP) without any predicted functional domain, suggesting that the legume preference of the pathogen is probably associated with unknown functions. This study forms the basis for further investigations into the mechanisms of interaction of *A. euteiches* with legumes.

## 1. Introduction

The *Aphanomyces* genus belongs to the order Saprolegniales, which includes filamentous eukaryotic pathogens that are encountered in different terrestrial and aquatic ecosystems [1,2]. This genus includes about 45 species that infect plants, animals (fish, crustaceans) and agricultural crops [1,3,4,5,6]. Thus, it is of great interest to study *Aphanomyces* to reveal the evolutionary mechanisms that allow host adaptation. *Aphanomyces euteiches* is a soil-borne phytopathogenic oomycete with a wide spectrum of leguminous hosts and affects legumes such as pea, lentil and alfalfa [1,7,8]. Due to its soil-borne nature and the production of oospores that stay viable for up to 10 years in the soil, long-term crop rotation has so far been the measure used to avoid *A. euteiches* infection [1]. *A. euteiches* induces root rot, which causes seedlings’ damping-off, yield decrease or even the death of the plant. This oomycete is highly virulent, mainly due to the production of a large number of motile biflagellate zoospores, which can move in liquid film in the soil and infect a whole field in a short time, leading to a complete loss of yield [2]. Several *A. euteiches* “pathotypes and/or races” have been identified based on their virulence on various legume hosts, such as pea or alfalfa [9,10,11]. Legume resistance against the pathogen is quantitatively mediated by diverse quantitative trait loci (QTL) that target different stages in the lifecycle of the pathogen [12,13]. However, the molecular basis for the adaptation of *A. euteiches* to various host legumes remains to be clarified.

Filamentous eukaryotic plant pathogens such as oomycetes secrete myriad proteins called effectors that modulate the host physiology and immune responses and enable parasitic infection [14,15,16,17]. Therefore, genomics studies on oomycetes involve exploring the organization and constitution of the complete set of secreted proteins defining the secretome [18,19]. This identification is facilitated by the fact that in eukaryotes, most of the secreted proteins follow the general secretory pathway, via a small N-terminal amino acid sequence known as the signal peptide [20]. In addition, oomycete effectors can be predicted due to the presence of a conserved amino acid motif generally in the Nter region. This motif has been detected in the RxLR and Crinkler families, which include hundreds of members [21,22,23,24,25]. Oomycete-secreted proteins include catalytic and non-catalytic proteins that function in ways that alter the plant cell wall, metabolism, nucleic acid integrity, signaling cascade, RNA-silencing and immune responses [15,26,27,28,29,30,31,32,33,34]. Thus, the prediction of the secretome opens up a new research area targeted toward understanding how secreted proteins function within the host cell to promote disease and thereby help the pathogen to adapt to the host.

Long-read-based sequencing technology routinely allows to obtain reads over 10 kb in length and significantly outperforms the Illumina short-read sequencing, where only reads of up to 600 base pairs can be obtained [35]. Using this advanced technique, numerous resequencing studies on filamentous pathogenic eukaryotes such as fungi and oomycetes have been undertaken. Long-read sequencing allows to obtain a continuous assembly, which can help to resolve localization of repeated regions and to improve gene annotations, notably for large families of genes such as effectors, which are often located in repeat-rich regions [36]. One example is the resequencing of the oomycete *Phytophthora parasitica* that helped to identify a new class of repeats corresponding to satellite DNA, which could not be identified in a short-read-based assembly [37]. Using long-read technology has helped to resolve the organization of repeated pathogenicity loci in the wheat fungal pathogen *Pyrenophora tritici* [38]. Resequencing a genome using long-read technology can also help to unravel the secretome, as demonstrated by the identification of a biosynthetic cluster of secondary metabolites that take part in the infection process of the fungal phytopathogen *Colletotrichum higginsianum* [39]. Further to this, in *Puccinia triticina*, genome resequencing allowed the prediction of a variety of new effectors [40].

In our previous study, we reported for the first time the 57 Mb genome assembly of the *A. euteiches* ATCC201684 strain based on a combination of Illumina and Roche 454 sequencing technologies. We estimated genome size of 61 Mb, predicted 19,548 genes and provided the first overview of the *A. euteiches* genome [41]. A comparative analysis with Illumina sequenced the crayfish pathogen *Aphanomyces astaci*, the saprotroph *Aphanomyces stelattus* and other well-studied oomycete phytopathogens such as *Phytophthora* was performed. The analysis detected a large set of Crinklers (CRNs), as well as a new family of secreted proteins called SSPs (small secreted proteins) unique to the *Aphanomyces* genus [41]. These SSPs are less than 300 residues in length and do not contain any predicted functional domain. This suggests that *Aphanomyces* spp. may develop its own original mechanism to achieve host invasion, one that is different from that of other oomycetes, such as the potato late blight oomycete *Phytophthora infestans*.

In this work, we first aimed to produce a long-read-based genome assembly of the *A. euteiches* ATCC201684 strain, generating a high-quality assembly in terms of contiguity and completeness. We identified 23,027 genes, of which 1515 (~6,5%) were further predicted to encode secreted proteins, and resolved the repeated regions. Long-read sequencing allowed us to provide the first-ever mitochondrial genome assembly for this plant pathogen. Four additional *A. euteiches* strains collected in different geographical areas and speciated to different legumes were subjected to Illumina sequencing. Through comparative analysis, we identified the core, accessory and specific secretomes of *A. euteiches*. We found that all the secretomes were closely related with respect to the functional classes of secreted proteins, but the ‘non-core’ secretomes were highly enriched in SSPs. This finding suggests that legume preference is linked to variation in the secretome content that has an unknown function. This work provides a useful resource for further studies on *A. euteiches*-plant interactions. Moreover, as the only plant pathogenic Saprolegniales species with a high-quality genome assembly, our work is also important to gain an increased understanding of oomycetes’ pathogenicity.

## 2. Materials and Methods

### 2.1. Aphanomyces ssp. Growth and DNA Preparation

*A. euteiches* ATCC201684, RB84, MF1, NC1 and Ae109 strains were grown for four days in a liquid YG medium (2.5% yeast extract, 5% glucose) at 23 °C in the dark. Mycelia were harvested and DNA extracted from ground mycelia using Macherey-Nagel Nucleobond RNA/DNA for the ATCC201684 strain and using our previously reported protocol for the other strains [41].

### 2.2. Genomes Sequencing, Assembly and Annotation

The preparation of all the libraries and sequencing was performed at the GeT-PlaGe core facility in Toulouse, France (https://get.genotoul.fr/en/). For ATCC201684, the library preparation was carried out according to the “Shared Protocol-30 kb Template Preparation/BluePippin Size Selection System”. A NanoDrop100 spectrophotometer (QIAGEN, Frederick, MD, USA), a Qubit (Life Technologies, Carlsbad, CA, USA) and a Fragment analyzer (AATI) were used to analyze the purity, integrity, quality and concentration of DNA. Genomic DNA was further purified using a BluePippin DNA size selection system (Sage Science, Beverly, MA, USA). Sequencing using PacBio SMRT technology to obtain long-reads was performed at the GeT-PlaGe core facility using 10 SMRTcells on a PacBio RSII Instrument. The raw sequencing reads were evaluated for their GC content distribution, quality distribution, base composition and average quality score at each position, and stored in NG6 [42]. The reads were de novo assembled using the RS Hierarchical Genome Assembly Process in SMRT analysis version 2.3.0 software (Pacific Biosciences, Menlo Park, CA, USA). For the RB84, Ae109, MF1 and NC1 strains, DNA-seq libraries were prepared using an Illumina TruSeq DNA v.2 Library Prep Kit following the manufacturer’s instructions. An Agilent Bioanalyzer was used to assess the quality of the libraries, and the libraries were quantified by qPCR using the Kapa Library Quantification Kit. DNA-seq experiments were performed on an Illumina HiSeq2000 Sequencer using a paired-end read-length of 2 × 100 pb with the HiSeq v.3 Reagent Kit at the GeT-PlaGe core facility in Toulouse. All the reads were quality-checked and stored in NG6 [42]. The reads of Ae109, RB84, MF1 and NC1 were assembled using MaSurCa [43] and the assembly metrics were calculated using assemblathon_stats.pl script (http://korflab.ucdavis.edu/datasets/Assemblathon/Assemblathon2/Basic_metrics/assemblathon_stats.pl).

Proteomes were predicted using the Augustus-based Braker 2 pipeline [44] trained with predicted proteins from a previous assembly, and RNASeq reads of zoospores and mycelium from *A. euteiches* [41]. Proteomes predictions were benchmarked using BUSCO software with the Stramenopiles reference gene set [45]. Repeated sequences were *de novo* identified using the RepeatModeller pipeline and the repeated sequence was then masked in the genome using RepeatMasker 4.1.0 (http://www.repeatmasker.org).

Satellite DNA was predicted using the bioinformatics algorithm as described in a previous publication [37]. Tandem Repeat Finder [46] was used to find tandemly repeated sequences in the genome; the resulting sequences were sorted by size to keep the ones with sizes from 100 bp to 500 bp. The monomeric sequences were blasted [47] against the genome (-perc_identity 85 -qcov_hsp_perc 90). The monomeric sequences that occurred less than 100 times were excluded from the analysis. Blastclust pairwise analysis was used to cluster monomeric sequences (identifying at 75%) to form families. Sequences of families were loaded into CLC Main Workbench software (Qiagen) to obtain the consensus sequence of the family. Sequences were aligned using ClustalW and the Neighbor-Joining method was used to build and visualize the phylogenetic tree in CLC Main Workbench software. All the data were stored in AphanoDB in August 2021 (https://www.polebio.lrsv.ups-tlse.fr/aphanoDB/) [48].

### 2.3. Mitochondrial Genome Assembly

A random subsample of 10% of self-corrected PacBio reads was used in the perl-based software Organelle_PBA (https://github.com/aubombarely/Organelle_PBA) as described in [49] to generate a circular contig. The *Aphanomyces astaci* mitochondrial genome (NCBI RefSeq: NC_032051, [50]) was used as the reference. Gene annotation was done on the MITOS webserver in May 2021 (http://mitos.bioinf.uni-leipzig.de) with further manual curation. The contig was visualized using the OGDRAW webtool [51].

### 2.4. Functional Characterization of Genome

Predicted proteins were assigned to protein families (PFAMs) and GO terms using InterProScan [52]. Secreted proteins were identified as reported in [53] using SignalP v.5 [54]. Proteins predicted with a transmembrane domain using TMHMM 2.0 [55] were excluded.

### 2.5. Comparative Analyses

The whole-genome alignment of the new and existing assemblies was performed with the LAST algorithm (http://last.cbrc.jp) and visualized in the D-genies tool (http://dgenies.toulouse.inra.fr). The protein predictions were compared with OrthoFinder v.2.5 using the DIAMOND engine for a sequence similarity search [56], and then they were visualized as a Venn diagram using the Bioinformatics & Evolutionary Genomics Venn diagram custom draw tool (http://bioinformatics.psb.ugent.be/webtools/Venn/). Based on the OrthoFinder results, the secreted proteins of the five strains were divided into three groups: core—from orthogroups containing orthologs of all five strains; accessory—from orthogroups containing orthologs of 2–4 strains; singletons—from orthogroups containing proteins without orthologs in other strains. For comparative studies, proteomes of *Phytophthora infestans* T30-4, *Saprolegnia parasitica* CBS223-65 and *Aphanomyces invadans* NJM9701 were downloaded from the FungiDB repository [57] and functional characterization of proteins was performed using the same procedure as for *A. euteiches.*

### 2.6. Small, Secreted Proteins’ (SSPs’) Prediction and Classification

To identify the SSPs, we selected secreted proteins of less than 300 amino acids in size. Proteins with a predicted functional domain were excluded based on previously reported InterProScan (v.5.48) results [52] using the following applications: TIGRFAM, PANTHER, Pfam, PIRSF, PRINTS, ProSitePatterns, ProSiteProfiles, SMART and SUPERFAMILY. To calculate the amino acid enrichment, we determined the distribution of amino acid frequency in the total proteome and calculated the third quartile of each amino acid (e.g., 75% of the total proteome has a lower share of the amino acid). We set a cutoff of 1.5× (third quartile) as enrichment. The phylogenetic tree was based on multiple alignments of SSPs from all the analyzed strains using Clustal Omega on the EBI website (https://www.ebi.ac.uk/Tools/msa/clustalo/). The tree was imported into iTOL software for visualization (https://itol.embl.de/).

## 3. Results

### 3.1. Genomic Features of the A. euteiches ATCC201684 Strain Defined by PacBio Technology

**Figure 1:**
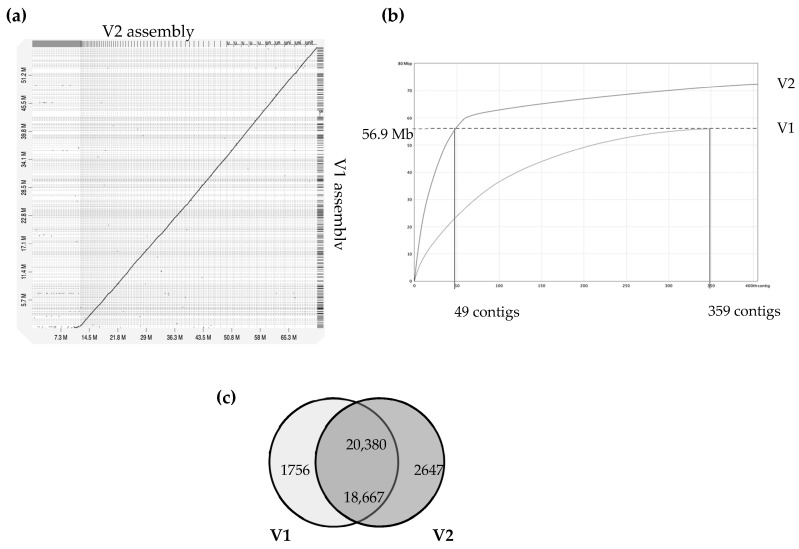
Comparison of version 1 and version 2 of *A. euteiches* genome assembly (ATCC201684 strain). A previous study using Illumina/454 technologies allowed the V1 assembly of the *A. euteiches* genome to be obtained [41]. This study provides the V2 assembly, obtained using long-read PacBio technology. (**a**) Dot-plot of pairwise whole-genome alignment of V1 and V2 assemblies. Note the additional sequence consisting of smaller contigs in V2 as compared to the V1 assembly. (**b**) Plot describing distribution of contigs by length for V1 and V2 assemblies. The 49 largest contigs of V2 cover the full size of the V1 genome. Additional contigs are present in V2. (**c**) Venn diagram of pairwise protein comparison of V1 and V2 (% ident > 75%, coverage > 75%). Shared part represents number of pairwise hits from V2 (upper number) and V1 (lower number).

The *A. euteiches* ATCC201684 strain was originally isolated from infected pea in Denmark, and in a previous report, we described how we generated a first genome assembly (version 1, V1) based on short-read sequencing technologies [41]. In this work, we aimed to improve the pathogen reference genome sequence using PacBio long-read technology to facilitate comparative genomic studies. Ten SMRT cells on PacBio RSII generated 1,414,231 total reads with a genome coverage of 146X and an average read length of 10.5 Mb. This resulted in a 72 Mb genome assembly (Table 1), the size of which was larger than the V1 assembly of 57 Mb (for an estimated genome size of 61 Mb), obtained by combining Illumina and 454 technologies. The GC content of around 47% was similar to the previous assembly. We improved the contiguity, as demonstrated by the big increase in N50 (N50 of 0.275 vs. 1.005 Mb). We generated a similar number of contigs, mainly because we obtained additional sequences present in rather small contigs (50–100 Mb in average; Figure 1). Indeed, we detected that the 49 largest contigs of V2 cover the full size of the V1 assembly, as depicted in Figure 1b, showing that a long-read assembly allows the detection of new contigs with putative new coding sequences. We identified that the additional V2 assembly contigs are distinct from the contigs aligned with the V1 version, with a lower gene content per Mb (329 vs. 112) and a higher percentage of repetition (16.8 vs. 64.6). The proteome was predicted by incorporating available expression data on *A. euteiches* previously generated by RNASeq technology [41,58]. We checked the completeness of the genome assembly using a Benchmarking Universal Single-Copy Ortholog (BUSCO) [45] based on 100 highly conserved Stramenopile proteins (100 proteins, odb10 dataset) and identified 98% complete genes, 2% fragmented genes and zero missing (Table 1). The two fragmented genes were EF-hand domain and mitochondrial-processing peptidase subunit beta, both of which harbor catalytic domains.

A total of 23,027 genes from the *A. euteiches* V2 (this study) were identified (Table S1a), representing an increase of 2404 genes when compared with V1. Comparison of proteomes from both assemblies showed that over 20,000 proteins from V2 (88.5%) had protein models identical (identifying at ≥ 75%) to those in V1. In our work, 2647 new protein models were predicted and 1756 protein models from V1 were not supported in V2 (Figure 1c). Among the new set of predicted proteins, only around 25% (i.e., 647 proteins) harbor a predicted PFAM domain mainly related to nucleic acid interactions, such as ‘integrase’, ‘endonuclease’, ‘transposase’, ‘RNAse’, ‘Helicase’ and ‘CENPB DNA-binding’ (Table S1b).

All the data were stored in an updated version of the AphanoDB database in August 2021 (https://www.polebio.lrsv.ups-tlse.fr/aphanoDB/) [48]. This resource is dedicated to “omics” studies on the *Aphanomyces* genus and provides tools such as a genome browser, gene annotation facilities and Basic Local Alignment Search Tool (BLAST) to facilitate analysis [48].

### 3.2. Repeat Content and Satellite DNA in A. euteiches ATCC201684

**Figure 2:**
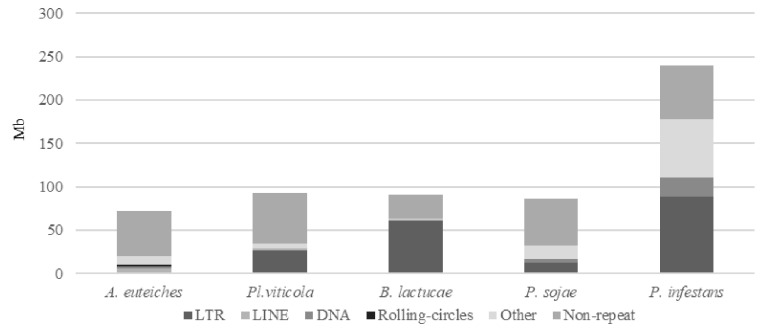
Repeats and non-repeated sequence distribution in plant pathogenic oomycete genomes as compared to *A. euteiches*. Three plant pathogenic oomycetes (*Plasmopara viticola, Bremia lactucae, Phytopthora sojae*) sequenced with long-read technology and the *P. infestans* genome were used to compare repeats and non-repeated sequences’ distribution within the legume pathogen *A. euteiches* ATCC201684. Families of repeats are indicated with a grey color. Data are available in ST1c-d.

We identified that close to 25% of the whole genome sequence of *A. euteiches* was represented as repeated sequences (Table S1c). This high level of repetition was not detected in our previous study where we used an Illumina/454 assembly [41]. As depicted in Figure 2, the most abundant repeat types were the LINEs repeats, which accounted for 3.7 Mb, and the LTR elements, which accounted for 2 Mb. To compare the levels of the different repeats in *A. euteiches* with those in other oomycetes, we selected three plant pathogenic oomycete genomes sequenced with long-read technology (*Plasmopara viticola* [58], *Bremia lactucae* [59], *Phytophthora sojae* [60]) and also included the *Phytophthora infestans* genome sequence [21]. When compared with the other oomycetes, the proportions of the different repeat types differed in *A. euteiches*, with it having the smallest size of repeated sequence and the largest portion of LINEs. Using the rolling-circles mechanism, we found that 1.5 Mb of the repeated sequences were predicted to be replicated. This has not been detected in other oomycetes (Figure 2, Table S1d).

**Figure 3:**
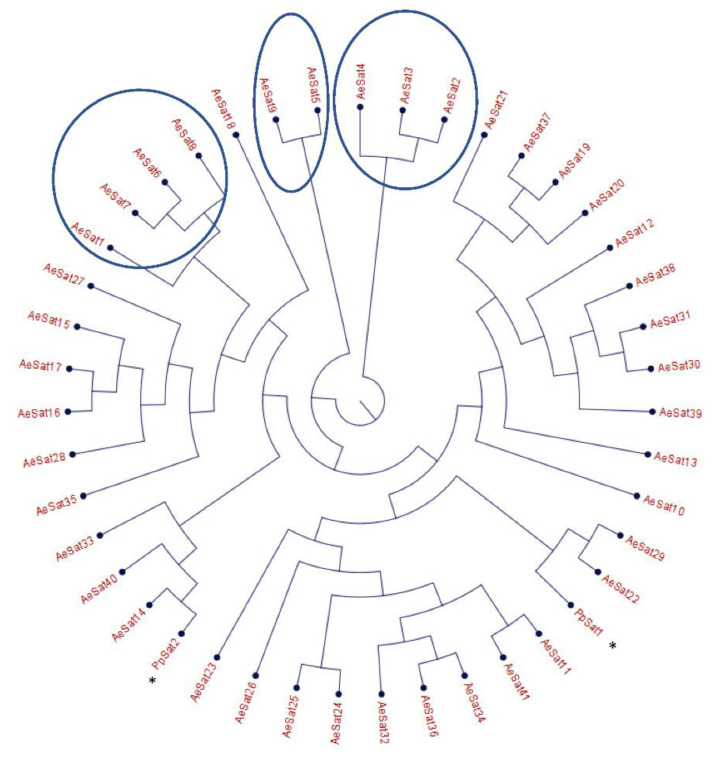
Phylogenetic tree of satellite DNA families from *A. euteiches*. Phylogenetic tree of the 41 satellite DNA families from *A. euteiches*, including the DNA satellite from *P. parasitica* * (PpSat1 and PpSat 2 [37]). HC = high number of copies of DNA satellite families (>1000 copies in the genome).

The large proportion of repeated regions in the *A. euteiches* genome prompted us to look for putative tandemly repeated sequences varying in size, copy number and sequence conservation, which could correspond to putative satellite DNA. The latter was recently reported following genome resequencing by long-read technology of the oomycete *Phytophthora parasitica* [37]. Based on the report by Panabières et al. [37], we searched for tandemly repeated sequences with monomer sizes ranging from 100 to 500 bp. The monomeric sequences were used to perform BlastN searches against the *A. euteiches* genome to identify the number of occurrences in the genome; the ones with more than 100 occurrences were kept for further analysis. We used blastclust pairwise comparison to group the tandem repeats into clusters based on sequence similarity. Our analysis revealed 41 families (AeSat) of tandem repeats matching satellite DNA criteria (Table S1e). As previously described for *Phytophthora*, the GC content might vary significantly between satellite DNA families, from 39% to 63% for the *A. euteiches* genome. The number of copies varied from 106 to more than 3000 for the four most-represented AeSat (i.e., AeSat1–AeSat4). In these regions, no coding sequences were identified, but for ten AeSat, we identified similarities with known transposable elements (TE), such as DNA/Harbinger, DNA/Helitron, NonLTR/Tad1, LTR/Gypsy and IntegratedVirus/DNAV (Table S1e). Sequence homologs of the two abundant families PpSat1 and PpSat2 from *P. parasitica* [37] were identified in *A. euteiches*. To unravel the relationship among satellite DNA families, a phylogenetic tree was constructed including PpSat1 and PpSat2 from *P. parasitica* [37] (Figure 3). The topology of the tree suggests diversification in AeSat families, with high copy number (i.e., >1000) families clustered into a central location group.

### 3.3. Mitochondrial Genome of A. euteiches ATCC201684

**Figure 4:**
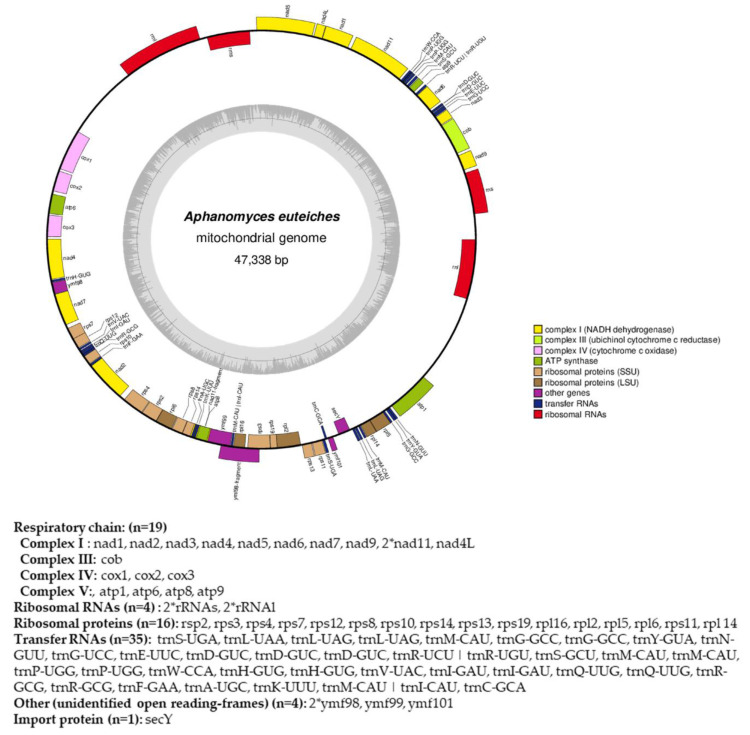
Circular map of the mitochondrial genome of *A. euteiches***.** Protein coding genes, tRNA and rRNA are shown on the outer colored ring. Genes encoded on both strands are listed below. The inner ring shows the GC density.

From the obtained long-read sequences, we assembled the mitochondrial genome (mtDNA) of the *A. euteiches* ATCC201684 strain. As depicted in Figure 4, the circular genome is 47 kb in size and is predicted to encode 40 protein-coding genes, 4 ribosomal RNA genes and 35 tRNA genes (Table S1f). The genes are encoded by both strands. As previously reported for mitogenomes of oomycetes [61], a bias in A/T usage is observed for the GC content around 22%. Using the available mitochondrial genome information (Table S1g), we compared the mitogenome content of *A. euteiches* with those of two other Saprolegniales (*Aphanomyces astaci* and *Saprolegnia ferax*) and one Peronosporale (*Phytophthora infestans*). The mitogenome size of *A. euteiches* is similar to that of the other Saprolegniales and larger than that of Peronosporale (e.g., 39.8 kb for *P. infestans vs.* 46.9 kb), as previously reported [50,62]. The gene content of mtDNA from *A. euteiches* is similar to those in other Saprolegniales and Peronosporales. A duplication of ribosomal RNA is observed in the Saprolegniales when compared with *P. infestans* (4 vs. 2). A secY gene that encodes for the central subunit of the secretory channel SecYEG, which enables the secretion of proteins across a membrane in their unfolded version, is present in the mitogenome of *A. euteiches* as it is in the other oomycetes.

### 3.4. Functional Annotation of A. euteiches ATCC201684 Genome

**Figure 5:**
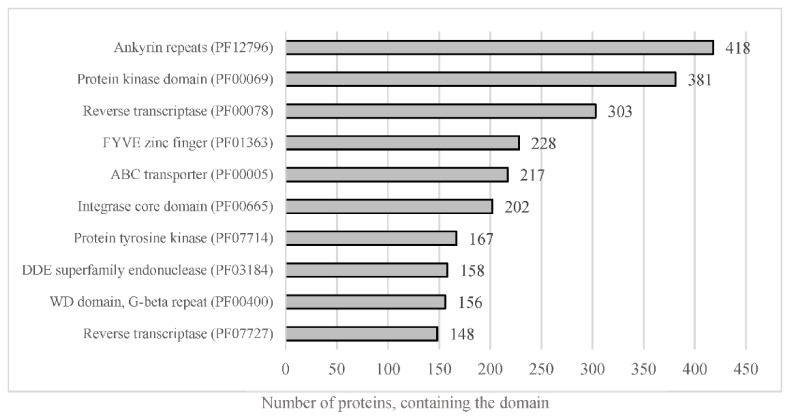
Representation of the most abundant PFAM predicted domain in the proteome of *A. euteiches*. When considering the number of proteins containing the most-present PFAM domains in total proteome, of the 23,027 total predicted proteins, around 56% harbor a PFAM domain.

The whole predicted proteome of *A. euteiches* was searched for the presence of PFAM domains. In total, 12,978 predicted proteins (56%) were found to harbor a PFAM domain (Table S1a). The most frequent were proteins containing Ankyrin and WD40 repeats, protein kinase domains and ABC-transporters, as shown in Figure 5. The genome of *A. euteiches* is also characterized by a large set of proteins containing domains related to nucleic acids such as the FYVE zinc finger, endonuclease and reverse transcriptase. We then searched the whole-proteome for putative secreted proteins, to predict the *A. euteiches* secretome. We identified 2106 proteins with a signal peptide (~9% total proteome) using SignalP V5 [54]. Among these, 1515 do not have a predicted transmembrane domain (6.5% of the proteome) and 591 contain at least one transmembrane region (TM; 2.5 % of the proteome). We annotated the CRN effectors as reported by [21] using the hmm-profile of the conserved N-ter domain constructed on CRN genes from the V1 assembly of the *A. euteiches* genome [41]. We predicted 234 CRN effectors, with less than 4% harboring a predicted signal peptide (i.e., 8 CRNs; Table S1h). In accordance with the V1 genome assembly and transcriptomic analyses, we did not detect any RxLR coding genes in *A. euteiches*. Among the secreted proteins without a predicted functional domain, we identified 568 small secreted proteins (SSP) with sizes of less than 300 amino acid residues and without any functional domains, as detected by an InterProScan search. This expands the original SSP repertoire detected in the V1 version of the genome. To confirm the putative role of SSPs as effectors, we performed an analysis using EffectorP v2. Around 80% of the SPPs (453 proteins) were predicted as effectors, among which 78% (351 proteins) were predicted as cytoplasmic effectors, 19% (88 proteins) as apoplastic effectors and 3% (15 proteins) as apoplastic or cytoplasmic effectors (Table S1i).

### 3.5. Secretome Features of A. euteiches ATCC201684 Strain

**Figure 6:**
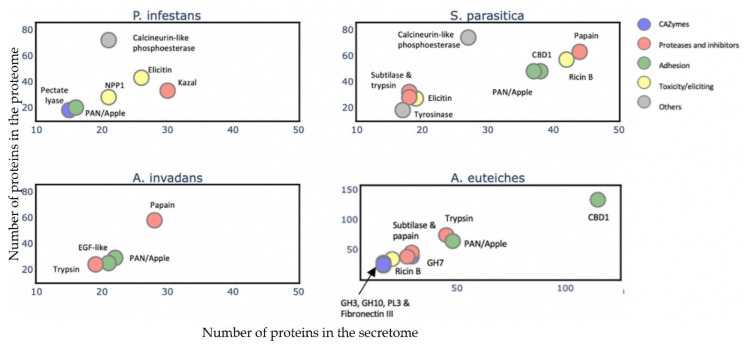
Comparison of *A. euteiches* secretome content with three other oomycetes. PFAM domains were searched in secreted proteins of two plant pathogens *Phytophthora infestans* (Peronosporale) and *Aphanomyces euteiches* (Saprolegniale) and two fish pathogens *Saprolegnia parasitica* and *Aphanomyces invadans* (Saprolegniale). Five classes of PFAM-containing proteins were identified. A PFAM domain present in more than 15 secreted proteins per class is represented as a colored circle and its name indicated above the circle. Arrow indicates overlaying circles. All selected PFAM domains were significantly enriched (Fisher’s exact test, p<0,05) with an enrichment coefficient (predicted secreted *vs.* expected secreted) over five for each class.

To gain an overview of the *A. euteiches* secretome’s composition and compare it with that of the other oomycetes, we identified and annotated the secretomes of two Saprolegniales, namely, the fish pathogens *Aphanomyces invadans* and *Saprolegnia parasitica*, and the distant Peronosporale plant pathogen *Phytophthora infestans* (Table S2a–c). We searched for the most represented PFAM-containing proteins and divided them into five classes (>15 proteins per class, Table S2d). The carbohydrate-active enzymes (CAZymes) class included secreted proteins that play a role in polysaccharides’ degradation in the host as well as in remodeling the oomycete cell wall. The protease and inhibitors class included secreted proteolytic proteins that take part in the alteration of the host immune system and in protecting against host lytic enzymes. The adhesion class comprised secreted proteins with adhesive capacity to different substrates such as cellulose and chitin and/or catalytic activity. The toxicity/elicitors class corresponded to secreted proteins that are known to trigger a host response or to be toxic to the host cells (i.e., elicitins, NLPs). The class labeled ‘others’ included all other secreted proteins known to play a role in host interaction (i.e., calcineurin-like phosphoesterase, tyrosinase). CRN and SSP families were not included in the analysis due to the absence of PFAM domains in a large majority of these proteins. Figure 6 presents the PFAM-containing proteins in the whole-proteome in each class with respect to the number that is expected to be secreted (Table S2k). We observe that a common trait of the oomycetes’ secretome is the ‘proteases and inhibitors’ class, which presents the most-enriched terms in Saprolegniales and Peronosporales with respect to the total protein content, but this is not the case for *A. euteiches*. The ‘adhesion’ class is a common trait among the four oomycete species, with an enrichment of PAN-Apple-containing proteins within this category such as the CBEL cell wall glycoprotein of *Phytophthora* sp., which contributes to plant adhesion and the cell wall architecture [63]. Overall, we notice that the phylogenetically distant *P. infestans* presents a distinctly different secretome profile compared to Saprolegniales species, characterized by the enrichment of toxic/elicitors proteins and protease-inhibiting Kazal domains. The Saprolegniales species, despite their different host preferences (plants vs. fish), show common features in their secretome profile such as enrichment in both adhesion domains and in several proteolytic domains. *A. euteiches* and *S. parasitica* secretomes seem more diverse in terms of their PFAM domains related to the different classes, as compared to *A. invadans*, and present an increase in proteolytic enzymes included in the ‘protease and inhibitor’ class. The *A. euteiches* secretome is also highly enriched in carbohydrate-binding proteins, especially with respect to proteins containing cellulose-binding domains (CBM1); this high number is a distinguishing feature that stands out when compared to the other species. Finally, the secretomes of plant pathogens share the CAZymes class as a unique and common feature.

### 3.6. Illumina Sequencing of Four Strains of A. euteiches with Differential Legumes Preference

To decipher the putative variability in legume pathogenicity genes within the *A. euteiches* lineage, we performed whole-genome sequencing of four strains of *A. euteiches* using Illumina technology. We selected strains with varied geographical origins and distinct host-spectra within legumes (Table 2). The RB84 strain, as the reference ATCC201684 strain, was isolated in Europe from pea fields and belongs to pathotype I according to pathogenicity tests performed with various genotypes of pea [64,65]. Both strains have a broad spectrum of hosts within the legumes but have a preference for pea [66]. The Ae109 strain, which was originally isolated from a pea field in the US and was subsequently shown to also be virulent in alfalfa, belongs to pathotype III. The MF1 and NC1 strains isolated in Wisconsin and North Carolina (US) vary in aggression and belong, respectively, to Race 1 and Race 2 of *A. euteiches* [52]. Race 1 is relatively widespread throughout the alfalfa-producing region in the US (Wisconsin and Minnesota) and Race 2 appeared in the 1990s after alfalfa failed to resist Race 1. Race 2 is considered to be more virulent than Race 1 and its prevalence is reported in US alfalfa fields and represents around 45% of all strains, with Race 1 representing 11% [67,68]. All the strains have been used in the implementation of GWAS on pea, or on the legume model *Medicago truncatula*, to characterize the genomic loci controlling resistance to *A. euteiches* [13,69,70].

We obtained four draft assemblies of 52–59 Mbp in size and with N50 values of 11 to 29 kbp (Table 3). The genomes had a GC content of about 47%, similar to the reference ATC201684 strain. Although the higher number of contigs in the NC1 strain suggests a slightly more fragmented assembly when compared to the three other strains, the level of genome completeness obtained using BUSCO analysis is similar for all the strains. We annotated around 22,000 protein-coding genes/strain except for the NC1 strain, which had 19,911. The proportion of secreted proteins is similar to that in the ATCC201684 reference strain and varies from 5.9% to 6.3% of the total proteome size (Table S3a–f). 

**Figure 7:**
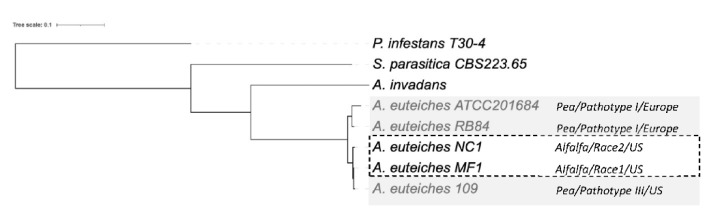
Phylogenetic relationship between the six sequenced *A. euteiches* strains. The maximum likelihood tree was built using Species Tree from All Genes (STAG) method from OrthoFinder. *A. euteiches, S. parasitica* and *P. infestans* were used as outgroups of different degrees of relativeness. MC1 and NC1 were identified as race 1 and race 2 (hatched square) based on their virulence on the Saranac and WAPH-1 alfalfa genotypes, while the ATCC201684, RB84 and Ae109 strains (grey square) were included in the ‘pathotype’ based on their aggressiveness against a pea collection. The geographical origins of the strains are indicated.

Next, we examined the phylogenetic relationship between the strains. We performed an OrthoFinder search to identify orthogroups (OG) of the proteins, to construct a Species Tree from all Genes (STAG) [74]. To identify the positions of newly sequenced strains within the oomycetes, we used the sequences of the two Saprolegniales fish pathogens (*Aphanomyces invadans* and *Saprolegnia parasitica*) and also included the Peronosporale plant pathogen *Phytophthora infestans* (Figure 7). The five strains of *A. euteiches* were grouped and formed a clade together with *A. invadans* within the Saprolegniales. Within the *A. euteiches* lineage, two subgroups were detected: one corresponding to European strains isolated from pea fields (ATCC201684 and RB84) and the other including strains isolated in the US (MF1, NC1 and Ae109), with the position of the NC1 strain being distinct within the subgroup.

### 3.7. Comparative Analysis of Functional Domains in Secretomes of A. euteiches Strains

**Figure 8:**
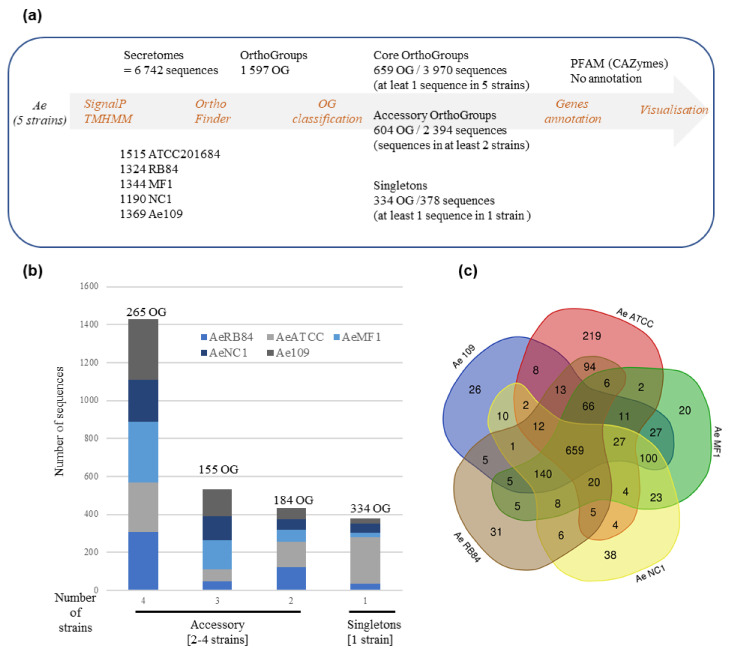
Comparative analysis of secretome of five *A. euteiches* strains with different host preference and geographical origins. (**a**) Pipeline used for comparative analysis of *A. euteiches* strains. (**b**) Distribution of sequences by *A. euteiches* strain (color-coded) found in accessory and singleton orthogroups. Number of orthogroups (OGs) is indicated. (**c**) Venn diagram depicting number of OGs per *A. euteiches* strain.

To check if the variation in the secretome content could be correlated to the host legume preference, we searched for shared and accessory secreted proteins in the five strains. To this end, the five secretomes were combined (totaling 6742 secreted proteins) and clustered using OrthoFinder. Proteins from the secretome were assigned to 1597 OrthoGroups (OG; Figure 8a, Table S3g). We defined a ‘core’ set of 659 OGs, where each OG had sequences from all the five secretomes analyzed, totaling 3970 secreted proteins. The accessory secretome was defined as OGs, which had proteins from two to four strains and comprised 604 OGs that encoded 2394 secreted proteins. A set of 378 secreted proteins in the form of ‘singletons’ clustered into 334 OGs with sequences from a single strain. Around 58% of the accessory secretome was shared by the four strains and less than 15% was specific to one strain (Figure 8b). A closer view of the OG distribution (Figure 8c) identified around 100 shared accessory OGs for the US strains (MF1/NC1/Ae109) and 94 for the European strains (ATCC201684/RB84). At the host level, 13 OGs were shared by the ‘pea’ strains (Ae109/ATCC201684/RB84) while 23 were shared by the ‘alfalfa’ strains (MF1, NC1) (Table S3h).

**Figure 9:**
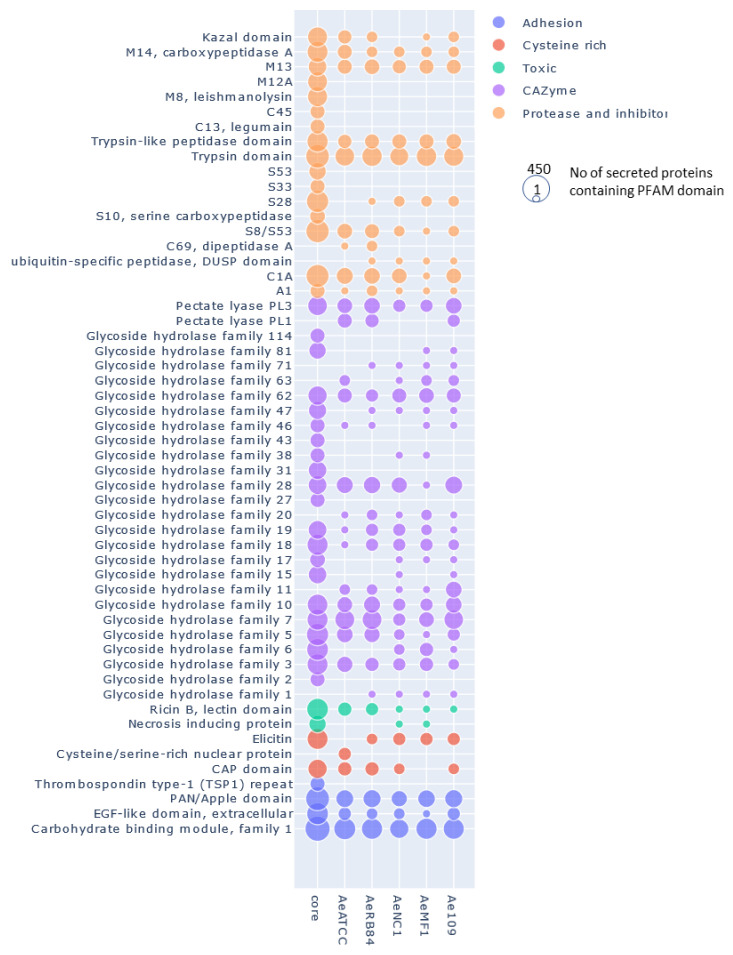
Distribution of secreted proteins containing a PFAM domain in the core and accessory secretome of *A. euteiches*. The first ‘core’ column represents the number of secreted proteins containing PFAM domains found in core orthogroups; the other columns represent the numbers of accessory and singleton orthogroups. The size of the circle corresponds to the log10 + 1 number of secreted proteins with a PFAM domain.

We then searched for the main functions that were present in the core and the accessory secretomes of *A. euteiches* by grouping the secreted proteins, based on their predicted PFAM domains, into classes as described above, except for the ‘toxic/elicitor’ class that we split into ‘toxic’ and ‘cysteine-rich’ classes for better visualization (Figure 9, Table S3i). CRNs and SSPs were not considered in this analysis either due to the absence of a predicted signal peptide or a PFAM domain. Around one-third of the secreted proteins of the core secretome (1186 of 3970) were predicted to be enzymes with activity against peptides (proteases) or cell-wall components (CAZymes).

In Figure 9, where the number of secreted proteins containing PFAM domains in the core (left column) and accessory secretomes (right columns) is shown, proteases can be seen to be largely distributed in the core secretome. The principal members of the core secretome are found to be families of metalloproteases M12A (astacin), M13 and M8 (leishmmanolysin), the serine proteases S10 (carboxypeptidase), S8/S53 (subtilisin) and S28 and the trypsin families. Cysteine protease families have a lower representation except for the C1A family (papain-type), while the C69 and C19 families are absent from the core secretome. We noticed the presence of Kazal serine protease inhibitors, which are known to be involved in the pathogenicity of *Phytophthora infestans* [75]. Cell wall-degrading enzymes, illustrated by CAZymes, constitute another major trait of the core secretome of *A. euteiches*. The most representative CAZymes families are present, including enzymes implicated in plant cell-wall degradation, such as polygalacturonases (family GH28) and cellulases (family GH5, GH6 and GH7), except for GH11 (xylanase), GH20 (hexosaminidase), GH63 (α-glucosidase) and PL1 (pectin lyase). We confirmed the presence of GH62 (α-l-arabinofuranosidase), not reported in other oomycetes except in *S. parasitica* [41,76]. In addition to the large proportion of ‘ricin’ and ‘necrosis-inducing’ domains that are known to trigger cell necrosis [77,78,79], the core secretome contains adhesive and cysteine-rich proteins involved in oomycetes’ pathogenicity, such as elicitin and CBM1 [30,80,81], or sterol binding CAP proteins that have recently been reported to be virulence factors in animal and plant pathogenic fungi [82].

In the ‘non-core’ secretome, the large majority of the secreted proteins displayed a predicted function that was already detected in the ‘core’ secretome. This is illustrated with S8/S53 proteases, GH62 or PL3 for CAZymes and PAN/Apple or CBM1 for adhesion (Figure 9), depicting the variation in the structural organization of the secreted proteins. We see that the five strains display species-specific proteins in the ‘accessory’ secretomes, as exemplified by the CAZymes GH11 and GH20. However, the NC1 strain contains no specific GH 46 (chitosanases) or Kazal-protease inhibitor and the MF1 strain is devoid of specific CAP secreted proteins. The difference between the European (ATCC201684/RB84) and the US (MF1/NC1/Ae109) ‘accessory’ secretome is the presence of specific C69 peptidases (present in European strains and absent in US ones) and GH6 and GH17 (cellulase) secreted proteins (absent in European strains and present in US ones).

### 3.8. Small Secreted Proteins (SSPs) with Unknown Function as Legume Adaptation Proteins

**Figure 10:**
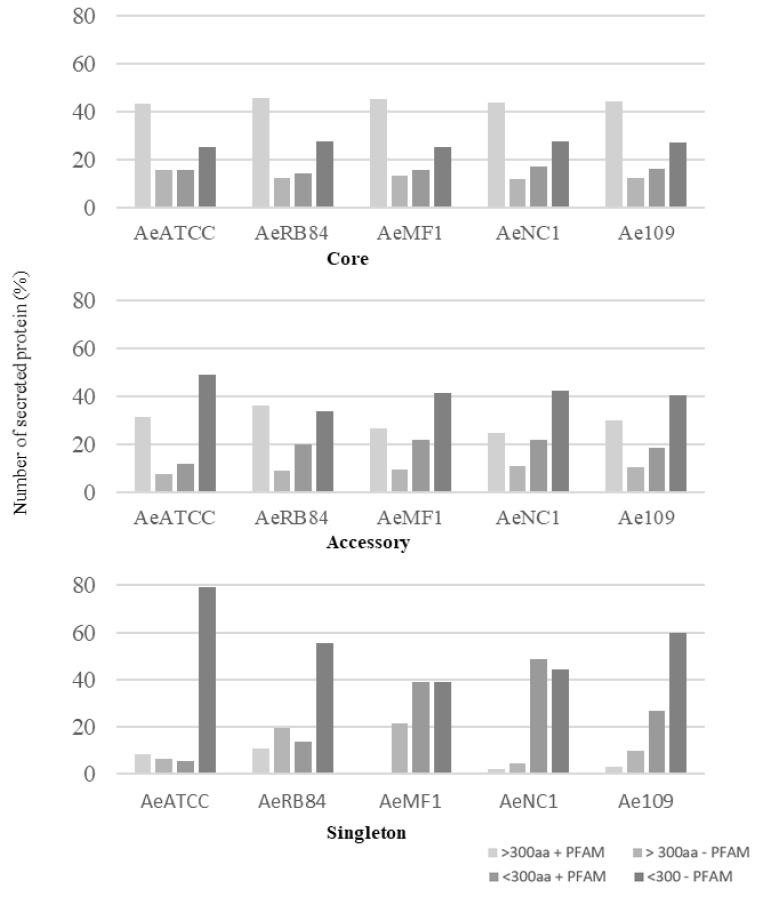
Size distribution of secreted proteins in the core, accessory and singleton secretomes of *A. euteiches* spp. *A. euteiches* spp. secreted proteins with (+) or without (−) a predicted PFAM domain are classified in two categories based on their size: larger than 300 amino acids (>300 aa) or less than 300 residues (<300 aa).

In the core genome, around 60% of the secreted proteins match a PFAM domain, while the accessory and singleton secretomes are characterized by a large representation of secreted proteins without any functional annotation (Figure 10, Table S3a–d).

**Figure 11:**
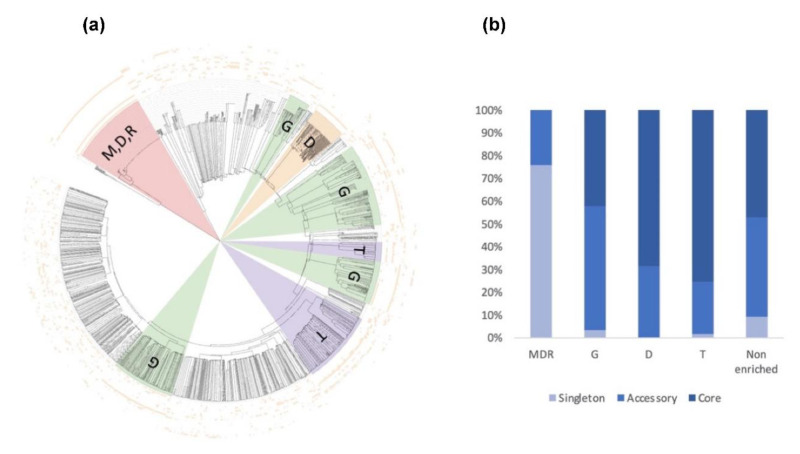
Consensus tree and class distribution of small, secreted proteins (SSP) within five strains of *A. euteiches*. (**a**) UPGMA consensus tree of 2010 SSPs from five strains of *A. euteiches* (RB84, Ae109, MF1, NC1, ATCC201684) based on whole protein amino acid sequence alignment using ClustalOmega (inner circle). Outside circles represent amino acid enrichment with one amino acid per circle (color = enriched, blank = non-enriched value). Colored ranges represent SSP classes with similar amino acid enrichment patterns. Green = G-rich, orange = D-rich, purple = T-rich, pink = MDR-rich. (**b**) Distribution of SSP classes (G-rich, D-rich, T-rich, MDR-rich, non-enriched) between core, accessory and singleton secretomes (in percent) of *A. euteiches*.

Among these, SSPs are less present in the ‘core’ secretome (44% for SSP vs. 58% for the total secretome) and are widely distributed in the ‘accessory’ and ‘singleton’ secretomes of the five strains. The higher occurrence of SSPs in ‘accessory’ and ’singleton’ secretomes of *A. euteiches* as compared to the core proteome indicates that these secreted proteins could participate in deciding legume preference. The task was then to identify classes within SSPs to find the corresponding structural regularities. The SSPs were first examined for the presence of conserved motifs or domains, as previously reported for RxLR and CRN effectors of oomycetes [21,24]. No conserved pattern was defined but we observed amino acid-enriched protein sequences in the SSPs. We consider as ‘enriched’ an amino acid with a 1.5-fold higher frequency than the third quartile value in the whole proteome of the strain. When plotting the amino acid enrichment on a phylogenetic tree of *A. euteiches* SPPs from the five strains, we see that clustered SPPs correspond to G-rich, T-rich and D-rich secreted proteins and account for 40–50 % of the total SSP set (Figure 11a).

Oddly, the long-read sequenced pea strain ATCC201684 is characterized by a large set of MDR-rich SPPs, not represented in the other Illumina-sequenced strains. These specific SSPs are tandemly repeated in the genome of the ATCC strain, while only one copy is detected in the Illumina sequenced strain, except for NC1, thus probably explaining the few sequences detected in the other *A. euteiches* species. While the distribution of SPPs within the five strains is almost similar, without considering the atypical MDR-rich class, the core secretome of *A. euteiches* contains T- and D-rich SSPs in contrast to the ‘non-core’ secretome characterized by G-rich SSPs (Figure 11b). This particular distribution of enriched-SSP suggests that they probably participate through an unknown function in the adaptation of *A. euteiches* to its environment.

## 4. Discussion

The mechanisms of pathogen adaptation to quantitative resistance in plants are largely unknown. In this paper, taking advantage of the availability of several *A. euteiches* strains adapted to pea or alfalfa, we investigated genetic variations that could be correlated with host preference, using a comparative genomics approach.

To obtain a high-quality 71 Mb genome assembly, we first re-sequenced, using PacBio, the ATCC201684 *A. euteiches* strain that we previously sequenced with a combination of Illumina and 454 technologies [41]. In this work, we improved the assembly and identified a large number of repetitive sequences as well as the presence of satellite DNA, as previously reported for the *Phytophthora parasitica* genome when re-sequenced by the long-read approach [37]. In addition, we assembled the first mitochondrial genome (mtDNA) for a Saprolegniales plant pathogen. The mitochondrial eukaryotic genome performs key functions in cells, such as the synthesis of nucleotides, and also contains the machinery for oxidative phosphorylation and electron transport, which are required for the production of energy [61]. The circular mitochondrial genome of *A. euteiches* is 47 kb in size. This size is similar to the genome size reported for other Saprolegniales such as *Aphanomyces astaci* and *Aphanomyces invadans* (49 kb) [50] and is different from those of Peronosporales (38 kb [83]) and Pythiales (55 kb [84]). This work reveals that the mitochondrial gene contents of plant and animal pathogenic Saprolegniales are very similar. In addition, the availability for the first time of the *A. euteiches* mtDNA offers us the possibility of developing new markers [83] and enables the detection of the pathogen with greater sensitivity [85] for root rot diagnostics.

A total of 23,027 protein-coding genes were identified in the V2 assembly of the *A. euteiches* genome, which is a significantly higher number than in V1. Among the newly predicted proteins present in the V2 assembly, 44% do not possess a putative functional PFAM domain, while the rest harbor PFAMs related to nucleic acid modification (i.e., integrase, endonuclease, transposase, etc.). Overall, the proteome exhibits numerous ankyrin repeats and WD-40 repeats. The ankyrin repeat is one of the most frequently observed amino acid motifs in protein databases, probably because this protein-protein interaction motif is involved in a diverse set of cellular functions [86]. Similarly, in eukaryotes, WD-40 repeat proteins generally mediate supramolecular interactions and participate in the assembly of complexes involved in different cellular processes [87]. These features may explain the large representation of ankyrin and WD40 repeats in the *A. euteiches* proteome. The expansion of the kinome (a set of protein kinases) observed in the *A. euteiches* genome has already been detected in the fish oomycete pathogen *Saprolegnia parasitica*, where these proteins are presumed to act as cell surface receptors [88]. The genome of *A. euteiches* encodes over 200 ABC transporters, a large proportion of which are commonly present in oomycetes [89]. Unlike fungi, oomycetes, in general, including *A. euteiches*, do not possess a set of detoxifying enzymes; notably, they are missing P450 enzymes. Therefore, the ABC transporters might counteract antimicrobial compounds, such as flavonoids [90]. The large repertoire of ABC transporters can also improve resistance to synthetic chemicals (e.g., fungicides), which complicates the application of chemicals to protect crops [91].

The secretome of plant-associated eukaryotic filamentous organisms such as fungi and oomycetes corresponds to secreted proteins that alter the environment to acquire nutrients or modify the host to facilitate invasion (effectors). Here, we predicted 1515 proteins, which corresponds to 6.5% of the *A. euteiches* ATCC201684 whole proteome. While we detected a larger set of Crinklers and SSPs in V2, we ascertained the absence of putative RxLR, which is predominant effectors in Peronosporales [92]. By looking for the major secretome components in *A. euteiches* and three other oomycetes, we confirmed the previous observation that the *A. euteiches* secretome is distinct from that of the animal pathogenic *Aphanomyces* [41] in harboring a large set of enzymes implicated in plant cell-wall deconstruction. The *A. euteiches* secretome is highly distinct from the one predicted in the plant pathogen *P. infestans*, which preferentially contains necrosis-inducing molecules (i.e., elicitin, NPP) and protease inhibitors (i.e., Kazal). Taken together, the PacBio long-read assembly of the ATCC201684 strain thus provides a high-quality reference genome for the *Aphanomyces* genus.

*A. euteiches* was initially considered as a pathogen of pea [6], but now we know that it can infect alfalfa, clover, dry beans and lentils, though not lupine or soybean [6–13]. To probe the molecular basis underlying the legume preference of *A. euteiches*, we compared the draft genomes of four strains of the pathogen (RB84, Ae109, MF1, NC1) from different geographical areas (Europe, US) with different pathogenicity traits on pea and alfalfa. Our phylogenetic analysis revealed that the strains MF1 and NC1 are closely related and are slightly distant from Ae109. The US strains are distant from the European strains (RB84, ATCC201684), suggesting an early divergence. The content of *A. euteiches* secretomes appears to be largely shared among all strains, with few gains/losses of secreted proteins. The ‘core’ secretome shares a number of properties, such as a large set of enzymatic proteins targeting carbohydrates and proteins. This set includes CAZymes, which are involved in the breakdown or binding of plant cell-wall carbohydrates such as cellulases and cellulose-binding proteins except for GH20 (hexosaminidase), GH63 (α-glucosidase) and PL1 (pectin lyase). We previously reported the enrichment of carbohydrate-binding and glycosyl hydrolases (GH) in the ATCC201684 strain [41,93], and the current work highlights the fact that the GH of CAZymes is a major trait of the *A. euteiches* ‘core’ secretome. This is in agreement with other studies that have identified a large repertoire of GH in oomycetes, with *Phytophthora* species tending to have the highest number as compared to other oomycete taxa [60,94,95,96]. Oomycetes produce CAZymes as a part of their arsenal for the supply of nutrition and to invade their preferential hosts. The GH repertoire may be linked to the oomycete lifestyle [19], with obligate biotrophic species having a reduced number and diversity of these proteins [97].

Another trait of the ‘core’ *A. euteiches* secretome is the presence of secreted cysteine and serine proteases except for C69 peptidases. The production of secreted proteases as a component of virulence in oomycetes that infect animals has received greater attention than plant pathogens. Secreted proteases have been proposed to be involved in the digestion of the host barrier, such as the crayfish cuticle in the case of *Aphanomyces astaci* [98], or the human epidermis in the case of *Pythium insidiosum* [99]. However, it is assumed that proteolysis of plant substrates is a strategy employed by plant pathogens during the infection process [100,101], meaning most of the proteases predicted in *Phytophthora sp*. contribute to pathogen virulence [102,103]. A previous study reported elevated pathogen-derived protease activity in pea tissues infected by *A. euteiches* (MN174 strain), whereas this activity was not required for saprophytic growth [104]. Thus, we propose that the core proteases of *A. euteiches* may act as potential pathogenicity factors.

The ‘non-core’ secretome of *A. euteiches* displays secreted proteins with functional activities reported in the ‘core’ secretome, depicting variation in the protein architecture rather than in its activity. Nevertheless, the two categories of CAZymes GH11(xylanases) and GH20 (hexosaminidases) are distinct characteristics of the ‘non-core’ secretome of *A. euteiches*. Xylanases are involved in the degradation of plant hemicellulose, and GH11 has been reported to participate in increasing the virulence of certain phytopathogenic fungi such as *Botrytis cinerea* or *M. oryzae* [105,106]. Interestingly, the soil-borne fungus *Verticillium dahliae* secretes a GH11(Vd424Y), essential for virulence, that targets the plant nucleus rather than the cell wall, suggesting an unknown function for fungal GH11 during host infection [107]. GH11s are not functionally characterized in oomycetes, but their presence in the ‘non-core’ secretome of *A. euteiches* in combination with GH62 (α-l-arabinofuranosidases) from the core secretome, probably provides improved access to the xylan backbone of plant hemicellulose, as reported for fungi [108]. GH20s, which degrade chitooligosaccharides (COS) into GlcNAc monomers [109], have been suggested as putative virulence factors shared by animal pathogenic oomycetes (Saprolegniales proteomes) but absent in phytopathogenic oomycetes [110]. The presence of chitooligosaccharides (COS) in the cell wall of *A. euteiches* [111,112] in contrast to other phytopathogenic oomycetes (Peronosporales), suggests the structural or protective role of GH20 for phytopathogenic *Aphanomyces* species.

Only minor content variations due to the presence/absence of secreted proteins allow us to distinguish the *A. euteiches* strains based on their geographical origin or legume preference. The US strains are characterized by the presence of specific cellulases (GH6, GH17), while the European strains harbor C69 peptidases. The presence of a specific set of secreted proteins could be related to the soil composition of the fields, with different histories of legumes in the US and Europe. The possibility of deciding the host preference based on compounds released by legumes roots has been suggested for closely related legume root-infecting *Phytophthora* species [113]. In addition, the use of partially resistant alfalfa cultivars in the US could also contribute to the diversity of *A. euteiches* as it could allow the selection of better-adapted strains such as NC1.

Finally, the ‘non-core’ secretome shows a higher abundance of small secreted proteins (SSP) with unknown functions as compared to the ‘core’ secretome. It is suspected that SSPs may play a role in the host adaptation of fungi but this aspect remains unclear [114]. However, their importance in the successful interaction with the host has been reported for various fungal symbionts and pathogens [114,115,116,117]. The presence of large variations in the gene number of SSPs within phytopathogenic fungi suggested that the molecular function of SSPs could be linked to the different infection strategies developed by such microorganisms [114]. In *Leptosphaeria maculans*, the expression of SSPs is regulated during plant infection and is also influenced by physical parameters such as the presence of antibiotics from prokaryotes in the rhizosphere [117]. This suggests that fungal SSPs, in addition to their role in plant interaction, may participate in ecological niche colonization by shaping plant-associated microbial interactions [118]. In a previous article, we reported the presence of SSPs in oomycetes [41]; here, we propose that SSPs may participate in determining the host preference. The classification of SSPs from *A. euteiches* into three groups based on their amino acid composition may help to unravel their unknown function. This is exemplified with AeSSP1256, a ‘core SPP’ from the G-rich class, which hijacks a *Medicago truncatula* RNA-helicase from its nucleic target and promotes *A. euteiches* roots’ infection [119].

In conclusion, this study provides a high-quality genome reference for *A. euteiches* at the nuclear and mitochondrial levels. Our comparative secretome analysis of *A. euteiches* species with different legume preferences mainly identified secreted proteins shared between species. The microbial molecular determinants of legumes’ preferences remain elusive, although this work sheds light on the presence of a certain degree of specificity at the level of SSPs’ repertoire.

## Figures and Tables

**Table 1:** Summary of *A. euteiches* ATCC201684 genome assemblies, annotation statistics and completeness evaluation.

Genome Version	Version V1 [Ref] Illumina/454	Version V2 (This Study) PacBio
Total contig length (Mb)	56.9	72
GC content (%)	47.69	47.27
Protein-coding genes	20,623	23,027
Average exons per gene	3.7	3.5
Mean gene size (kb)	1503	1447
N50 (kbp) N90 (kbp)	275,164 69	1,005,788 39
Gene density (nb genes/Mb)	343	320
Coverage	146x	148x
Number of scaffolds	349	420
BUSCO complete/ fragmented/duplicated /missing	83.1%/3.8% (Alveolata-Stramenopiles dataset)	98%/2%/0%/0% (Stramenopiles dataset)

**Table 2:** Origin and host spectrum of *A. euteiches* strains.

*A. euteiches* Strain	Isolated from	Origin	Host-Spectrum within Legumes *	References
ATCC201684	Pea	Denmark	Broad: pea, alfalfa	[41,71]
RB84	Pea	France	Broad: pea, alfalfa, bean, lentil, vetch	[66]
Ae109	Pea	USA	Narrow: pea, alfalfa	[9,70,72]
MF1	Alfalfa	USA	Narrow: alfalfa but not pea	[67,73]
NC1	Alfalfa	USA	Narrow: alfalfa but not pea	[10,73]

* All the strains can interact with the model legume *M. truncatula*.

**Table 3:** Assembly statistics of four near-complete *A. euteiches* genome sequences.

Description	NC1	MF1	RB84	109
Assembly size (Mb)	52.04	59.75	59.39	58.41
N50 (bp)	11.661	24.852	29.237	21.846
Scaffold count	15.477	7.808	7.095	6.495
GC content (%)	47.73	47,46	47.45	47.43
Protein-coding gene count	19.911	22.012	21.892	21.546
Number of secreted proteins (%)	1.190 (5.9%)	1.344 (6.1%)	1.324 (6%)	1.369 (6.3%)
BUSCO score for Stramenopiles (%) (complete/duplicated/ fragmented/missing)	100/0/0/0	98/2/0/0	97/0/0/3	100/0/0/0

## Data Availability

Assemblies, sequencing and annotation are available in the publicly accessible repository AphanoDB v2.0, updated in August 2021 (http://www.polebio.lrsv.ups-tlse.fr/aphanoDB/). The genome assembly of *A. euteiches* ATCC201684 (Illumina/545 combination) is also available via the EMBL|European Nucleotide Archive (ENA) (https://www.ebi.ac.uk/ena/browse) under study number PRJEB24016. For the other genome assemblies, data are currently under submission at ENA as follows: ATCC201684 (PacBio); PRJNA769534|MF1; PRJNA767766|NC1; PRJNA767769|RB84; PRJNA767773|Ae109; PRJNA767593.

## Supplementary Materials

The following are available online at https://www.mdpi.com/article/10.3390/jof8010088/s1, Table S1: *A. euteiches* (pea strain ATCC201684) genome annotation (PacBio); Table S2: Oomycete secretomes; Table S3: *A. euteiches* strains’ annotation (Ae109, RB84, MF1, NC1 strains, Illumina).
